# Supplementary material for: Wolves and dogs fail to form reputations of humans after indirect and direct experience in a food-giving situation
Source: PLoS One. 2022 Aug 17;17(8):e0271590. doi: 10.1371/journal.pone.0271590 (PMC9385025; doi:10.1371/journal.pone.0271590)
Supplement: S1 File — (DOCX) [file pone.0271590.s001.docx]

## **S1 File. Habituation and exceptions.**

Some animals were fearful of strangers, thus they had habituation sessions that were 10-15 minutes long before participating in the experiment, and for some of the animals that were fearful of sudden movements, the procedure was modified (see table below). During the habituation sessions, the partners acted neutrally and identically so the animals would not develop a bias for one person.

Only one dog (Panya) needed habituation sessions, in which the partners entered the test enclosure while Panya was inside with two trainers and a familiar pet dog for social support. This is not an unusual experience for the animals at the Wolf Science Center (WSC), as they have weekly pack visits, where they can interact with strangers voluntarily. For the wolves, the partners stood outside of their home enclosures and called their name and threw food through the fence. This did not influence the wolves’ experience in the experiment because this was like Session 1 of the study. Two wolves (Nanuk and Una) were still not habituated to the unfamiliar humans after eight sessions; thus, they were excluded due to time constraints.

**List of subjects that required habituation and exceptions.**

| Species | Animal | Extra information |
| --- | --- | --- |
| Dogs | Panya | 3 habituation sessions in Condition 1. In the choice tests, the partners knelt and looked down. |
| Wolves | Etu | Did not participate in behavioural tests with unfamiliar humans. |
|  | Kaspar | Did not participate in behavioural tests with unfamiliar humans. |
|  | Tekoa | Did not participate in behavioural tests with unfamiliar humans. |
|  | Nanuk | Excluded from Condition 1 because he showed signs of stress during the experimental procedure. 8 habituation sessions in Condition 2 – excluded |
|  | Una | Excluded from Condition 1 because she showed signs of stress during the experimental procedure. 8 habituation sessions in Condition 2 – excluded |
|  | Maikan | In the choice tests, the partners stood facing the fence and looked down. |
|  | Taima | 3 habituation sessions in Condition 2. In the choice tests, the partners stood facing the fence and looked down. |
|  | Kenai | 4 training sessions in Condition 1, where the partners walked up to the enclosure. 4 habituation sessions in Condition 2. In the choice tests, the partners stood facing the fence and looked down. |
|  | Wamblee | Tested in home enclosure (see below) |

Wamblee was tested in his home enclosure due to logistical problems. The wolf packs at the WSC regularly change home enclosures and at the time of testing, his home enclosure was 1,901 m^2^ in Condition 1 and 7,069 m^2^ in Condition 2. There are no small compartments in the home enclosures to act as an observer’s area, thus he observed from inside and the interactions took place outside the enclosure (see figure below).


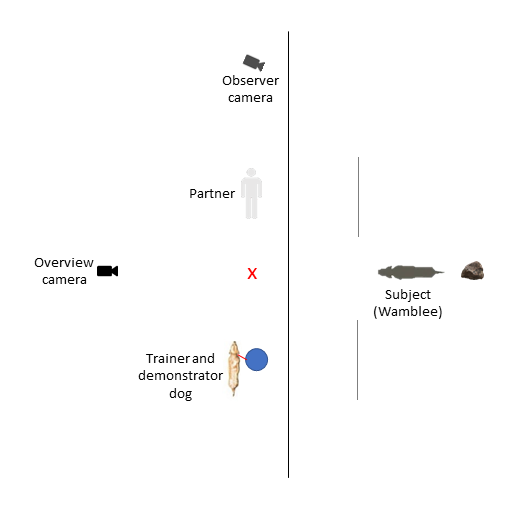


**Schematic depiction of the experimental setup for Wamblee in his home enclosure.**
