# Supplementary material for: Wolves and dogs fail to form reputations of humans after indirect and direct experience in a food-giving situation
Source: PLoS One. 2022 Aug 17;17(8):e0271590. doi: 10.1371/journal.pone.0271590 (PMC9385025; doi:10.1371/journal.pone.0271590)
Supplement: S1 Table — Estimates, standard error, confidence intervals, results of significance tests and minimum and maximum of model estimates derived after excluding individuals one at a time. (DOCX) [file pone.0271590.s002.docx]

**S1 Table. Results of the full model for the eavesdropping subset.** Estimates, standard error, confidence intervals, results of significance tests and minimum and maximum of model estimates derived after excluding individuals one at a time.

| **Term** | **Estimate** | ***SE*** | **95% *CI*** | | ***z*** | ***p*** | **Min** | **Max** |
| --- | --- | --- | --- | --- | --- | --- | --- | --- |
|  |  |  | **Upper** | **Lower** |  |  |  |  |
| Intercept | -1.209 | 1.677 | 2.028 | -4.9 |  |  | -1.952 | -0.158 |
| Species: Wolf^a^ | 0.134 | 1.582 | 3.393 | -3.174 | 0.085 | .933 | -0.809 | 1.176 |
| Single trial in Session 2^b^ | 2.119 | 2.678 | 7.957 | -2.891 | 0.791 | .429 | 1.077 | 3.386 |
| First trial of Session 3 | 0.700 | 2.669 | 6.355 | -4.552 | 0.262 | .793 | -16.272 | 2.255 |
| Condition: Experimental^c^ | -0.660 | 1.656 | 2.587 | -4.154 | -0.399 | .690 | -2.322 | 1.254 |
| z-transformed order | 0.341 | 0.336 | 1.069 | -0.289 | 1.015 | .310 | -0.143 | 0.515 |
| z-transformed attentiveness | -1.103 | 0.947 | 0.603 | -3.265 | -1.164 | .244 | -1.638 | -0.666 |
| Species × single trial in Session 2 | 1.631 | 2.191 | 6.312 | -2.595 | 0.744 | .457 | 0.851 | 18.72 |
| Species × first trial of Session 3 | 2.287 | 2.189 | 7.041 | -1.852 | 1.045 | .296 | 1.333 | 19.304 |
| Species × condition | 0.135 | 2.030 | 4.314 | -3.849 | 0.066 | .947 | -1.885 | 1.697 |
| Single trial in Session 2 × condition | 0.081 | 2.229 | 4.555 | -4.400 | 0.036 | .971 | -1.428 | 1.689 |
| First trial of Session 2 × condition | -0.763 | 2.613 | 4.409 | -6.299 | -0.292 | .770 | -17.545 | 15.339 |
| Species × single trial in Session 2 × condition | -4.644 | 3.068 | 1.142 | -11.13 | -1.514 | .130 | -22.147 | -3.055 |
| Species × first trial of Session 3 × condition | -0.692 | 2.988 | 5.339 | -6.741 | -0.231 | .817 | -17.033 | 16.081 |

Estimate, standard error, confidence intervals, results of significance tests (Wald’s *z* approximation) and the range of estimates derived after excluding individuals one at a time.
^a^Species: dog as reference level.
^b^Session 1 (baseline) as reference level.
^c^Condition: control as reference level.
