# Supplementary material for: Wolves and dogs fail to form reputations of humans after indirect and direct experience in a food-giving situation
Source: PLoS One. 2022 Aug 17;17(8):e0271590. doi: 10.1371/journal.pone.0271590 (PMC9385025; doi:10.1371/journal.pone.0271590)
Supplement: S2 Table — Estimates, standard error, confidence intervals, results of significance tests and minimum and maximum of model estimates derived after excluding individuals one at a time. (DOCX) [file pone.0271590.s003.docx]

**S2 Table. Results of the full model for the reputation-learning subset.** Estimates, standard error, confidence intervals, results of significance tests and minimum and maximum of model estimates derived after excluding individuals one at a time.

| **Term** | **Estimate** | ***SE*** | **95% *CI*** | | ***z*** | ***p*** | **Min** | **Max** |
| --- | --- | --- | --- | --- | --- | --- | --- | --- |
|  |  |  | **Upper** | **Lower** |  |  |  |  |
| Intercept | -1.088 | 0.522 | 2.573 | 0.125 |  |  | -1.831 | -0.806 |
| Species: Wolf^a^ | 1.127 | 0.616 | 0.097 | -2.639 | 1.830 | .067 | 0.838 | 1.790 |
| z-transformed trial | -0.190 | 0.421 | 1.594 | -0.649 | -0.451 | .652 | -0.543 | -0.027 |
| Condition: Experimental^b^ | 0.423 | 0.685 | 1.137 | -1.953 | 0.618 | .537 | 0.103 | 1.116 |
| z-transformed order | 0.017 | 0.182 | 0.386 | -0.441 | 0.094 | .925 | -0.036 | 0.081 |
| z-transformed attentiveness | -0.093 | 0.252 | 0.642 | -0.408 | -0.370 | .712 | -0.352 | 0.062 |
| Species × z-transformed trial | 0.163 | 0.529 | 0.992 | -1.707 | 0.308 | .758 | 0.007 | 0.517 |
| Species × condition | -1.002 | 0.746 | 2.714 | -0.619 | -1.344 | .179 | -1.497 | -0.639 |
| z-transformed trial × condition | 0.418 | 0.580 | 0.790 | -2.093 | 0.722 | .470 | 0.149 | 0.773 |
| Species × z-transformed trial × condition | -0.367 | 0.722 | 2.285 | -1.215 | -0.508 | .611 | -0.722 | -0.104 |

Estimate, standard error, confidence intervals, results of significance tests (Wald’s *z* approximation) and the range of estimates derived after excluding individuals one at a time.
^a^Species: dog as reference level.
^b^Condition: control as reference level.
