# Supplementary material for: Wolves and dogs fail to form reputations of humans after indirect and direct experience in a food-giving situation
Source: PLoS One. 2022 Aug 17;17(8):e0271590. doi: 10.1371/journal.pone.0271590 (PMC9385025; doi:10.1371/journal.pone.0271590)
Supplement: S3 Table — Estimates, standard error, confidence intervals, results of significance tests and minimum and maximum of model estimates derived after excluding individuals one at a time. (DOCX) [file pone.0271590.s004.docx]

**S3 Table. Results of the full model for the direct experience subset.** Estimates, standard error, confidence intervals, results of significance tests and minimum and maximum of model estimates derived after excluding individuals one at a time.

| **Term** | **Estimate** | ***SE*** | **95% *CI*** | | ***z*** | ***p*** | **Min** | **Max** |
| --- | --- | --- | --- | --- | --- | --- | --- | --- |
|  |  |  | **Upper** | **Lower** |  |  |  |  |
| Intercept | -0.495 | 0.256 | 0.053 | -1.072 |  |  | -0.621 | -0.291 |
| Species: Wolf^a^ | 0.006 | 0.331 | 0.702 | -0.701 | 0.019 | 0.985 | -0.202 | 0.132 |
| z-transformed trial | -0.210 | 0.257 | 0.299 | -0.837 | -0.816 | 0.415 | -0.295 | -0.139 |
| z-transformed order | -0.123 | 0.162 | 0.214 | -0.462 | -0.754 | 0.451 | -0.206 | 0.011 |
| Species × z-transformed trial | 0.185 | 0.332 | 0.983 | -0.462 | 0.557 | 0.578 | 0.060 | 0.329 |

Estimate, standard error, confidence intervals, results of significance tests (Wald’s *z* approximation) and the range of estimates derived after excluding individuals one at a time.
^a^Species: dog as reference level.
