# Supplementary material for: Wolves and dogs fail to form reputations of humans after indirect and direct experience in a food-giving situation
Source: PLoS One. 2022 Aug 17;17(8):e0271590. doi: 10.1371/journal.pone.0271590 (PMC9385025; doi:10.1371/journal.pone.0271590)
Supplement: S4 Table — Significant p values are in bold. (DOCX) [file pone.0271590.s005.docx]

#### **S4 Table. Results of the exact binomial tests for side and partner bias.** Significant p values are in bold.

| **Species** | **Subject** | **Number of trials** | **Side bias** | | **Partner bias** | |
| --- | --- | --- | --- | --- | --- | --- |
|  |  |  | **Number of successes (left)** | ***p*** | **Number of successes (generous)** | ***p*** |
| Wolves | Amarok | 30 | 10 | .099 | 12 | .362 |
|  | Chitto | 30 | 13 | .585 | 12 | .362 |
|  | Geronimo | 23 | 2 | **< .001** | 14 | .405 |
|  | Kenai | 27 | 12 | .701 | 18 | .122 |
|  | Maikan | 30 | 0 | **< .001** | 13 | .585 |
|  | Taima | 15 | 3 | **.035** | 9 | .607 |
|  | Tala | 28 | 11 | .345 | 21 | **.013** |
|  | Wamblee | 30 | 19 | .201 | 14 | .856 |
|  | Yukon | 30 | 9 | **.043** | 26 | **< .001** |
| Dogs | Enzi | 21 | 9 | .664 | 18 | **.001** |
|  | Hiari | 20 | 4 | **.012** | 9 | .824 |
|  | Imara | 30 | 9 | **.043** | 21 | **.043** |
|  | Layla | 30 | 14 | .856 | 23 | **.005** |
|  | Panya | 27 | 11 | .442 | 18 | .122 |
|  | Zuri | 27 | 10 | .248 | 12 | .701 |
